# Investigating Factors that Generate and Maintain Variation in Migratory Orientation: A Primer for Recent and Future Work

**DOI:** 10.3389/fnbeh.2016.00003

**Published:** 2016-01-21

**Authors:** Kira E. Delmore, Miriam Liedvogel

**Affiliations:** Behavioural Genomics Department, Max Planck Institute for Evolutionary BiologyPlön, Germany

**Keywords:** seasonal migration, next-generation sequencing, candidate gene, genomics, transcriptomics, epigenetics

## Abstract

The amazing accuracy of migratory orientation performance across the animal kingdom is facilitated by the use of magnetic and celestial compass systems that provide individuals with both directional and positional information. Quantitative genetics analyses in several animal systems suggests that migratory orientation has a strong genetic component. Nevertheless, the exact identity of genes controlling orientation remains largely unknown, making it difficult to obtain an accurate understanding of this fascinating behavior on the molecular level. Here, we provide an overview of molecular genetic techniques employed thus far, highlight the pros and cons of various approaches, generalize results from species-specific studies whenever possible, and evaluate how far the field has come since early quantitative genetics studies. We emphasize the importance of examining different levels of molecular control, and outline how future studies can take advantage of high-resolution tracking and sequencing techniques to characterize the genomic architecture of migratory orientation.

Considerable variation has been documented in the orientation of migratory routes (e.g., across years for individual wood thrushes, Stanley et al., 2012; within populations of humpback whales, Horton et al., 2011; and between populations of monarch butterflies, Altizer and Davis, 2010). Several lines of evidence, including quantitative genetic analyses and experimental approaches, suggest that this variation may be partially genetically determined, derived from standing genetic variation and controlled by a few genes of large effect (quantitative genetic analyses summarized in Pulido and Berthold, 2003; experimental approaches include displacement, Chernetsov et al., 2008; crossbreeding, Helbig, 1996; selection, Kent and Rankin, 2001; and common gardens, Plantalech manel-la et al., 2011). These approaches permit inferences on the heritability of traits but fail to provide details on the causative variants and genes. This limitation has prevented researchers from asking many key questions on the genetic basis of migratory orientation, such as: where are the causative variants located—do they cluster physically, are they in regulatory or coding regions? Is natural selection or drift responsible for their propagation? Are the causative genes in the same functional classes? Are the same genes involved across different groups (species, genera, families, classes)?

Information on the genetic basis of migratory orientation is relevant to many fields, including evolution (e.g., behavioral genetics, Hoekstra, 2010; microevolution, Rolshausen et al., 2009; and speciation, Irwin and Irwin, 2005) ecology and conservation (e.g., environmental contributions to variation, the establishment of conservation strategies, Faaborg et al., 2010a,b; Winkler et al., 2014; and pest management, Jones et al., 2015). In this perspective, we will: (1) describe molecular genetic techniques employed thus far to identify the causative variants and genes for migratory orientation (including potential pitfalls) and (2) highlight new and suggested techniques for future work on this topic. Several recent reviews have summarized the genetic basis of migratory orientation (e.g., Liedvogel et al., 2011; Brönmark et al., 2014; Liedvogel and Lundberg, 2014; Chapman et al., 2015). Accordingly, our aim is to stimulate future research in this area, not to discuss current understanding of the topic.

Our focus is on migratory orientation but we will include inferences from other traits as well; seasonal migration is a behavioral syndrome that incorporates several traits (e.g., the propensity to migrate, wing length, hyperphagia, Dingle, 2006). Many of these traits are correlated (Pulido and Berthold, 1998; Roff and Fairbairn, 2007) and thus may be controlled by the same regulatory switch. Alternatively, a change in one trait may necessitate a change in the others. It should also be noted, that many of these traits may use similar machinery (Merlin et al., 2009) and differences in orientation necessitate changes in the other traits (e.g., timing of arrival, staging sites, hyperphagia and moulting, Figure 2 in Piersma, 2011).

## Techniques Employed Thus Far

Efforts to identify variants and genes associated with migration began largely with candidate gene analyses, where researchers look for differences in either the sequence or expression of genes that influence a trait in one organism in another organism. For example, circadian rhythms interpret photoperiod and are key regulators of migratory timing (Gwinner, 1996). Accordingly, many studies have focused on conserved and well characterized genes that regulate biochemical oscillations with circadian cycles. One series of studies correlated allele frequencies in a polyQ domain of *Clock* with various photoperiod-entrained phenotypes. Many of these studies uncovered a regulatory role for *Clock* (e.g., migratory timing in Chinook and Pacific salmon, O’Malley and Banks, 2008; O’Malley et al., 2010; breeding phenology in blue tits and barn swallows, Liedvogel et al., 2009; Caprioli et al., 2012; and migratory propensity in the *Junco* genus and barn swallows, Peterson et al., 2013; Saino et al., 2015; timing of migration assessed via light-level geolocators Bazzi et al., 2015). Nevertheless, subsequent studies on different populations or taxa have failed to find similar associations (e.g., no correlation with latitudinal clines in bluethroats, barn swallows, *Tachycineta* swallows or three-spined stickleback, Johnsen et al., 2007; Dor et al., 2011, 2012; O’Brien et al., 2013; breeding phenology in great tits or barn swallows, Liedvogel and Sheldon, 2010; Dor et al., 2011; or migratory propensity in blackcaps, Mueller et al., 2011).

We should note, that *Clock* is predicted to regulate migratory timing and not orientation. Nevertheless, this gene is extremely well studied and thus we have used it here as an example. There is only one set of suggested candidates for migratory orientation: cryptochromes. These molecules have been discussed as putative light-dependent magnetoreceptors; the magnetic compass likely aids with migratory orientation and thus cryptochromes may be crucial for this behavior (reviewed in Liedvogel and Mouritsen, 2010). Differences in cryptochrome expression patterns between migratory phenotypes have been identified using real-time PCR (RT-PCR) in garden warblers (Mouritsen et al., 2004) and blackcaps (Fusani et al., 2014). Cryptochromes may also help regulate circadian feedback loops in molecular clocks, making them encouraging candidates for future work.

Candidate gene analyses have provided us with important inferences but are associated with a few drawbacks. First, as demonstrated with *Clock*, there are inconsistencies across studies, making it difficult to draw general conclusions from this work. Many of the candidates were also initially identified in model organisms that do not migrate (e.g., crytochromes were initially characterized in plants, *Arabidopsis*, Ahmad and Cashmore, 1993; *Chlamydomonas*, Small et al., 1995) and thus many candidates could be missing from our lists. Our lists are also restricted by a lack of knowledge concerning migration’s genetic basis, so that most of the focal loci are candidate genes for anticipated and preselected candidate traits. This point is especially true for migratory orientation (vs. for example timing or the propensity to migrate, where any circadian gene could be a candidate), where the only candidate genes to date are cryptochromes.

In response to these limitations, researchers have begun to expand beyond candidate gene analyses towards *de novo* discovery, employing reduced-representation techniques like amplified fragment length polymorphisms (AFLPs) and restriction site associated DNA (RAD) tag sequencing (Davey et al., 2011; Etter et al., 2011). Both methods use restriction enzymes to cut the genome into smaller fragments; AFLPs use presence-absence scores at cut sites and RAD tags use high-throughput sequencing around these sites. Two species of Atlantic eel breed in the Sargasso Sea but migrate along opposite coasts of the Atlantic. Gagnaire et al. (2009) identified 27 AFLP loci that distinguish these species; since migration is the main difference between these groups, genes in proximity to these loci may be associated with migratory differences.

By including information from all restriction enzyme cut sites, reduced-representation techniques capture a larger portion of the genome and do not require *a priori* knowledge of candidate genes. Nevertheless, these techniques are associated with their own set of drawbacks. To begin with, these markers are often anonymous (e.g., AFLPs cannot be easily mapped to a reference genome; RAD tags are often assembled *de novo*) and AFLPs are dominant markers that do not provide information on homozygosity/heterozygosity. Some RAD studies align their *de novo* identified markers to a reference but this reference is often for another organism. Accordingly, researchers have to assume the genome of their focal species is sufficiently similar to the reference (e.g., in gene order and karyotype). It should also be noted that until recently, very few genomes had been assembled for migratory species (e.g., the collared flycatcher genome was the first reference for a migratory bird and was not published until Ellegren et al., 2012). Similar to candidate gene analyses—this means that genes associated with migration are likely underrepresented. Finally, reduced-representation techniques are, by definition, still only surveying a subsample of the genome (i.e., variation at restriction enzyme cut sites). This restriction could be problematic if a few genes of large effect control migratory traits, as there would be less chance of a marker being linked to the causative variant.

Limitations of reduced-representation sequencing are illustrated well in a comparison between Ruegg et al. (2014) and Delmore et al. (2015); these authors sought to determine if candidates for migration were in genomic regions that differentiate inland and coastal Swaisnon’s thrushes. Ruegg et al. (2014) used RAD tags mapped to the zebra finch genome. Delmore et al. (2015) assembled a reference genome for the Swainson’s thrush and used whole genome shotgun (WGS) sequencing, where the genome is broken up by sonication and sequence data is obtained from all fragments within a specific size range (Medini et al., 2008; Metzker, 2010). These authors obtained opposite results: Delmore et al. (2015) found more migration candidates in differentiated regions than expected by chance while Ruegg et al. (2014) did not. This discrepancy is likely related to differences in resolution, with Delmore et al. (2015) having an order of magnitude more variant sites (SNPs), increasing their coverage of genes and allowing more fine-scale analyses. Delmore et al. (2015) also compared the number of SNPs they would have called if they had used the zebra finch genome and found an almost a twofold increase.

## New and Suggested Techniques

RAD tags and WGS sequencing are next-generation sequencing (NGS) tools; they rely on platforms like 454, SOLiD and Illumina that produce hundreds of thousands (to millions) of sequences in a single run (Medini et al., 2008; Metzker, 2010). These platforms have reduced the amount of time and money required for sequencing and their continued development will undoubtedly enhance our understanding of the genetic architecture of migratory orientation (Stapley et al., 2010). This suggestion was illustrated well by Delmore et al. (2015) and a few additional studies, including Zhan et al. (2014), where WGS sequencing was used to compare migratory and non-migratory monarchs using the recently assembled monarch genome (Zhan et al., 2011). These authors identified one region that differentiated migrants and non-migrants. This region included one gene (collagen IV alpha-1) that showed signatures of selection and is essential for muscle morphogenesis and function. The non-migratory haplotype was shared among all non-migratory populations suggesting it evolved from standing genetic variation.

The use of WGS sequencing for admixture mapping across migratory divides could be a particularly fruitful avenue for future research. Migratory divides are areas where populations breed adjacent to one another but use different migratory routes (e.g., Irwin and Irwin, 2005; Møller et al., 2011; Rohwer and Irwin, 2011). Hybrids zones often form at these divides and would be ideal for admixture mapping. Admixture mapping is the search for significant associations between phenotypes and genetic variants that makes use of recombination in natural hybrids (Buerkle and Lexer, 2008). This method has many benefits over traditional approaches, including quantitative trait loci (QTL) and association mapping. QTL analyses rely on lab crosses for mapping. Linkage disequilibrium (LD) is high in these crosses preventing fine scale mapping. Association mapping, on the other hand, uses variation within populations. LD is low in these populations allowing for fine-scale mapping but requiring the use of many markers. Admixture mapping falls between these two extremes, as it uses natural hybrid zones for mapping which often include both early (high LD) and late (low LD) hybrids (Buerkle and Lexer, 2008). By relying on natural hybrid zones, admixture mapping also precludes the necessity to generate crosses, which can be difficult in many systems. To the best of our knowledge, mapping studies for migration have been limited to salmonids and used association and QTL mapping (e.g., Hale et al., 2013; Hecht et al., 2013; Pearse et al., 2014).

NGS can also be used to compare gene expression patterns between distinct phenotypes. For instance, RNAseq uses NGS to obtain information on transcript content (sequence) and abundance (count). This technique has several benefits over traditional expression methods (e.g., RT-PCR, quantitative PCR and microarrays). These benefits have been reviewed extensively elsewhere (e.g., Wang et al., 2009; Vijay et al., 2013; Wolf, 2013) but include the fact that information on SNPs, transcript splicing, and allele-specific expression can be obtained with RNAseq and the quantification of abundance does not saturate at high levels of expression as with microarrays. In addition, microarrays often don’t exist for non-model organisms. Work conducted by McKinney et al. (2015) exemplifies the potential of RNAseq. These authors used RNAseq to quantify gene expression between trout produced from migratory vs. non-migratory parents. They identified several differentially expressed genes related to brain growth and development and obtained additional functional information by aligning these genes to RAD tags and QTL previously shown to be associated with migration.

McKinney et al. (2015) also used a pathway analysis to determine if there were biological functions or pathways that were enriched with differentially expressed transcripts. Similar analyses can be conducted using WGS sequencing; they are called gene ontology (GO) analyses and are used to identify enrichment of GO terms in, for instance, genomic regions that differentiate two phenotypes. Both of these techniques can be particularly helpful for expanding lists of candidates.

One of the ultimate steps to unambiguously identify genes associated with migratory orientation will require genetically disrupting them *in vivo* and observing their subsequent behavior. At present, there are a few different methods available for this work. For instance, knockdown mutants can be produced using RNA interference (RNAi), where RNA molecules with sequences complimentary to a candidate gene are introduced into a cell and activate RNAi pathways that inhibit the expression of that gene. In birds, these RNAi can either be delivered by lentiviral injections into specific structures (e.g., Haesler et al., 2007) or through germline transformations (Agate et al., 2009; Abe et al., 2015). Genome editing technologies using engineered endonucleases has also been employed to knock genes out (e.g., monarch butterfly, Merlin et al., 2013). Recently, there has been much interest in actually editing genes using CRISPR/Cas technology (Jinek et al., 2012). With this method, an RNA molecule complimentary to the candidate is designed and delivered with a Cas9 protein to the cell where it modifies the gene (adds, disrupts or changes the sequence). This method is more precise than the former methods, as only the target sequence is being edited (vs. inserting the gene at a random position). Regardless, all of these methods may ultimately rely on model organisms for the time being (e.g., monarch butterfly, Reppert et al., 2016; among birds the only successful transgenics are chicken, quail and zebra finches Scott et al., 2010).

So far we have focused on hard-coded changes in the DNA sequence that could account for variation in migratory orientation. Epigenetic gene regulation (e.g., DNA methylation, histone tail modifications, noncoding RNAs) could also be relevant, as considerable phenotypic plasticity has been documented in migratory traits (e.g., rapid evolution of new migratory routes and wintering areas, Berthold et al., 1992). DNA methylation has been the focus of much study; it usually involves a reduction in gene expression caused by the methylation of CpG sites. Methyl-sensitive AFLP (MS-AFLP) and reduced-representation bisulfite sequencing (RRBS) are two methods used to quantify this process (Schrey et al., 2013). MS-AFLP uses the AFLP protocol described above with a methyl-sensitive restriction enzyme that does not cut un-methylated CpG sites. RRBS uses a methyl-insensitive restriction enzyme and treats the resultant fragments with sodium bisulfite. This compound deaminates un-methylated cytosines, converting them to uracil. The fragments are sequenced with WGS and methylated CpG sites can be distinguished from un-methylated sites by the presence of cytosine at the cut site (vs. a thymine). RRBS is more expensive than MS-AFLP but—similar to RAD and WGS—is favored over anonymous AFLP markers that do not make use of NGS.

Work conducted by Baerwald et al. (2015) illustrates the potential of RRBS. These authors used RRBS to compare DNA methylation in migratory and non-migratory trout. Fifty-seven differentially methylated regions (DMRs) were identified; many of these encoded proteins relevant to the propensity to migrate (e.g., circadian rhythm, nervous system development, protein kinase activity). More than half of the DMRs were within or near CpG islands, suggesting that epigenetic modifications are influencing transcriptional activity of associated genes (CpG islands are believed to serve as sites for transcription initiation). In addition, close to half of the DMRs were not located in proximity to known genes, implicating trans-acting regulatory elements.

As a final point, we have focused on developments in molecular genetics in the current perspective, but in order to identify causative variants for migratory orientation it is equally important to obtain accurate information on the phenotype of individuals. Fortunately, developments in molecular genetics have been paralleled by advances in movement ecology. We outline a few of the recent developments in this field below but direct readers to recent reviews for complete information (Robinson et al., 2009; Chapman et al., 2011; McKinnon et al., 2013; Hedenström and Lindström, 2014; Deng et al., 2015).

In the context of insect migration, radar technologies including vertical-looking and harmonic radars are now capable of quantifying the flight behavior of known/named species (Chapman et al., 2011). Advances in acoustic technology have made it easier to track fishes on migration, with the miniaturization of transmitters making it possible to inject them into individuals using a syringe and needle, eliminating the need for surgery. These devices also capture data over much longer periods of time (Deng et al., 2015). A technique for tracking extremely small (mm-sized) aquatic organisms has also been developed. This technique makes use of fluorescent nanoparticles and multiple synchronized cameras to obtain information the movement of organisms and should be possible to apply to small land-based organisms as well (Ekvall et al., 2013).

All of the former techniques represent significant advances in movement ecology and will undoubtedly be important for studies of seasonal migration. Nevertheless, none of them allow for the acquisition of data from individual organisms over the entire annual cycle. The main methods for obtaining this individual data remain GPS and satellite tags and indeed these technologies have seen many developments as well, including reductions in size and increases in accuracy (Hedenström and Lindström, 2014). Nevertheless, these tags are still quite large, limiting their use to larger animals (turtles, large mammals and birds).

Arguably the biggest advance for tracking individuals over the entire annual cycle has been the miniaturization of light-level geolocators (McKinnon et al., 2013; Hedenström and Lindström, 2014). Light-level geolocators are archival tags that record light intensity. These devices are attached to animals on the breeding grounds and retrieved the following year when light intensity data are used to infer longitude and latitude for every day the device was on the bird. Light-level geolocators are now small enough to be fitted to songbirds, making them particularly relevant for the present perspective as there is likely a strong genetic component to migration in this group; they migrate alone and at night, precluding the option of learning their migratory routes from older individuals.

It could be very informative to repeat some of the original studies on migratory orientation using NGS tools and new methods developed in movement ecology. One set of experiments that would be exciting to repeat are those conducted Helbig (1991, 1994, 1996), who bred F1 and F2 hybrids between blackcap populations that form migratory divides. He assayed their orientation using Emlen funnels and observed that hybrids oriented in intermediate directions, suggesting this trait may be inherited additively. Some F2s oriented in directions more extreme than parental forms further suggesting that this trait may be controlled by a few genes of large effect. Delmore and Irwin (2014) repeated this work using geolocators and natural hybrids between inland and coastal Swainson’s thrushes. These authors observed that—as expected—hybrids took intermediate routes. Nevertheless, they only used three markers to identify hybrids; this study would be greatly improved by the use of NGS tools to genotype hybrids into specific classes (e.g., to compare the orientation of F1 hybrids to that of backcrosses).

## Conclusion/Outlook

We anticipate that work on the genetic basis of migration will benefit substantially from the advent of NGS and believe that migratory divides represent the perfect arena for studies focusing specifically on migratory orientation. In a time where there is considerable interest in genomics and transcriptomics, it is important to remember that epigenetics can also contribute to variation in traits and indeed, the rapid changes observed in migratory orientation may be facilitated by these modifications. A complete understanding of migration’s genetic basis will likely require integration across fields, not only with movement ecology but also with neurogenetics and neurosensory sciences. This suggestion derives from the fact that the main biological reference systems used by various taxa to orient during migration are celestial cues (sun, star) and the Earth’s magnetic field and each of these compasses relies on visual perception and neuronal integration. Eventually we hope that enough information will be available to not only answer the questions outlined at the start of our perspective but also to estimate the relative role of nucleotide sequences, gene expression and epigenetics in generating variation in migratory orientation. We encourage and look forward to applications of findings from this work to not only migration but also evolution, ecology and conservation.

## Author Contributions

KED and ML conceived and wrote this perspective together.

## Funding

Funding was provided by the Max Planck Society and NSERC.

## Conflict of Interest Statement

The authors declare that the research was conducted in the absence of any commercial or financial relationships that could be construed as a potential conflict of interest.
